# Involvement of miR-30a-5p and miR-30d in Endothelial to Mesenchymal Transition and Early Osteogenic Commitment under Inflammatory Stress in HUVEC

**DOI:** 10.3390/biom11020226

**Published:** 2021-02-05

**Authors:** Carmen Ciavarella, Ilenia Motta, Francesco Vasuri, Silvia Fittipaldi, Sabrina Valente, Daniela Pollutri, Francesca Ricci, Mauro Gargiulo, Gianandrea Pasquinelli

**Affiliations:** 1 Laboratory of Clinical Pathology, Department of Experimental, Diagnostic and Specialty Medicine (DIMES), St. Orsola-Malpighi Hospital, University of Bologna, 40138 Bologna, Italy; ilenia.motta2@unibo.it (I.M.); silvia.fittipaldi3@unibo.it (S.F.); sabrina.valente2@unibo.it (S.V.); daniela.pollutri2@unibo.it (D.P.); gianandr.pasquinelli@unibo.it (G.P.); 2 Surgical Pathology Unit, Department of Experimental, Diagnostic and Specialty Medicine (DIMES), St. Orsola-Malpighi Hospital, University of Bologna, 40138 Bologna, Italy; francesco.vasuri@gmail.com; 3 Immunohaematology and Transfusion Medicine Service, St. Orsola-Malpighi Hospital, 40138 Bologna, Italy; francesca.ricci@aosp.bo.it; 4 Vascular Surgery Unit, Department of Experimental, Diagnostic and Specialty Medicine (DIMES), St. Orsola-Malpighi Hospital, University of Bologna, 40138 Bologna, Italy; mauro.gargiulo2@unibo.it; 5 Subcellular Nephro-Vascular Diagnostic Program, Pathology Unit, IRCCS, St. Orsola-Malpighi University Hospital, Via Albertoni 15, 40138 Bologna, Italy

**Keywords:** endothelial to mesenchymal transition, osteogenic differentiation, vascular injury, inflammation, atherosclerosis, calcification, micro-RNA

## Abstract

The endothelial to mesenchymal transition (End–MT) can be associated with vascular calcification, by providing mesengenic progenitors. In this study, we investigated a link between End–MT and the osteogenic process and explored the involvement of miR-30a-5p and miR-30d as potential regulators of these processes. End–MT was induced in Human Umbilical Vein Endothelial Cells (HUVEC) through transforming growth factor-β1 (TGF-β1), TGFβ-3 and tumor necrosis factor-α (TNF-α), for 24 h and 6 days. End–MT mediators, mesenchymal and osteo/chondrogenic markers were analyzed through Real-Time PCR, immunofluorescence, flow cytometry and Western Blot. miR-30a-5p and miR-30d over-expression was carried out in HUVEC to explore their effects on End–MT and osteogenic differentiation. HUVEC at 24 h and 6 days gained mesenchymal morphology markers, including matrix metalloproteinase 9 (MMP-9), SLUG, VIMENTIN and α-smooth muscle actin (α-SMA), and a significant migratory potential, notably with TNF-α. After 6 days, the osteo/chondrogenic markers runt-related transcription factor 2 (RUNX-2) and SRY box transcription factor 9 (SOX-9) were upregulated. At this time point, miR-30a-5p and miR-30d decreased. Over-expression of miR-30a-5p and miR-30d affected End–MT mediators and the osteogenic potency in HUVEC, by reducing SLUG, VIMENTIN and RUNX-2. Our data suggest that End–MT represents a key link between inflammation and vascular calcification. Further, miR-30a-5p and miR-30d can regulate both the End–MT and the osteogenic processes, prompting future studies for exploring their potential use as therapeutic targets or biomarkers in vascular diseases.

## 1. Introduction

Endothelial cells (ECs) constitute the inner cell layer within the blood vessels and are primarily involved in vascular homeostasis, regulating blood flow, hemostasis, blood cells’ luminal adherence and vascular permeability [1]. The endothelial dysfunction is considered the triggering event in the initiation of atherosclerotic disease [2], and it is associated with the loss of the EC barrier, allowing the recruitment of monocytes and the infiltration of low-density lipoproteins (LDLs) within the intima. Oxidation of LDLs is followed by foam cell generation, a powerful stimulus initiating the inflammatory cascade. Smooth muscle cells (SMCs) are induced to migrate and proliferate into the intima, forming the major component of the atherosclerotic plaque. Arterial calcification is commonly associated with vascular disease, and currently the coronary calcification score is used to define the burden of atherosclerotic disease.

However, it is not clear whether SMCs are the only cells involved in the mineralization of the intima. Several studies have pointed out the presence of circulating osteogenic progenitors able to migrate into areas of vascular injury [2], while others reported the presence of resident mesengenic progenitors in the adult arterial wall [3,4]. One additional emerging mechanism is the occurrence of endothelial to mesenchymal transition (End–MT) [5]. In fact, ECs are highly plastic cells, able to de-differentiate into a multipotent mesenchymal progenitor, and End–MT is an essential step in cardiogenesis and vasculogenesis during the embryonic development [1,6]. In this process, ECs undergo molecular rearrangements, by losing their cobblestone morphology and tight junctions, and acquiring mesenchymal features, including elongated shape, migration, matrix remodeling abilities, as well as cytoskeletal alterations [5]. These structural and functional changes are accompanied by the downregulation of typical endothelial markers, i.e., CD-31, vascular endothelial cadherin (VE-cadherin) and the acquisition of mesenchymal markers, like fibroblast-specific protein (FSP) and α-smooth muscle actin (α-SMA). End–MT can be triggered by different factors, like the members of the transforming growth factor (TGF)-β family, which includes growth factors, bone morphogenetic proteins (BMP) and activins [7]. The binding between TGF-β members and relative receptors activates the receptor-regulated SMADs, which translocate to the nucleus and regulate downstream gene expression [8]. The occurrence of End–MT has already been described in systemic and organ-specific fibrotic diseases [9], pulmonary hypertension [10] and atherosclerosis [11], supporting the association between endothelial plasticity and vascular injury due to inflammation or surgical procedures. In atherosclerosis, End–MT can be one of the sources of mesenchymal progenitors, which critically drive the pathological remodeling of the vascular wall. In this regard, we previously demonstrated that MSCs derived from normal and atherosclerotic aortas have increased osteogenic potential when exposed to inflammatory conditions [12], hypothesizing a further mechanism contributing to plaque calcification. End–MT is modulated by different signaling pathways, transcription factors and microenvironment cues. Micro-RNAs (miRNAs) are small non-coding RNAs (18–24 nucleotides) that act as endogenous regulators of gene expression by binding to the 3′untranslated region (UTR) of the mRNA target. miRNAs can control different biological processes, like cell proliferation, differentiation and migration. It has been recently shown that miRNAs influence the endothelial cell plasticity by targeting transcripts involved in End–MT [13]. We recently discovered a differential deregulated miR-30a-5p and miR-30d signature in carotid artery plaques that was associated with distinct histological calcification patterns [14]. Interestingly, by using miRNA prediction target tools, we found End–MT mediators (VIMENTIN, Snail family transcriptional repressor-1 (SNAI1)) and regulators of the osteogenic and chondrogenic commitment (Runt-related transcription factor, RUNX-2; SRY box transcription factor, SOX-9) among miR-30a-5p and miR-30d predicted targets [15]. Further, miR-30a-5p was reported to downregulate epithelial–mesenchymal transition (EMT) [16], a process occurring during morphogenesis and oncogenesis in epithelial cell lines; this process, correlating with cancer progression and metastasis, is analogous to End–MT. Due to their regulatory role on different processes with relevance to the cardiovascular system, miRNAs have been proposed as potential biomarkers for staging cardiovascular diseases, for patient stratification, or as therapeutic targets. In this regard, literature studies performed in animal models showed that miR-21 inhibition was associated with reduced in-stent restenosis [17], whereas miR-29b inhibition reduced the growth of abdominal aortic aneurysm [18]. However, the introduction of miRNAs in the clinical practice is challenging for many technical issues, including the need for standardized protocols for miRNA analysis. Moreover, miRNA-based therapeutic approaches require an accurate evaluation of miRNA signaling mechanisms; indeed, miRNAs are broadly expressed among tissues and their manipulation can lead to side effects [19]. For this reason, unveiling the biology of miRNAs and their role in disease models would be crucial to their utilization for diagnostic and therapeutic purposes in the field of cardiovascular diseases.

In order to investigate the link between End–MT and plaque calcification, we firstly established an in vitro model of End–MT driven by inflammatory soluble factors: in addition to the well-known TGF-β1, we included TGF-β3, belonging to the same family, and Tumor Necrosis Factor-α (TNF-α), as an inflammatory cytokine mimicking the atherosclerotic milieu. Then, we explored the miRNA biology during End–MT and osteogenic differentiation, by inducing the ectopic expression of miR-30a-5p and miR-30d in this cell model.

## 2. Materials and Methods

### 2.1. Cells and Culture Conditions

Human Umbilical Vein Endothelial Cells (HUVEC; Lonza, Basel, Switzerland), were cultured in Dulbecco’s Modified Eagle’s Medium (DMEM, Sigma-Aldrich, St Louis, MO, USA) enriched with 10% Fetal Bovine Serum (FBS, Sigma-Aldrich, St Louis, MO, USA) and 1% penicillin/streptomycin (Sigma-Aldrich, St Louis, MO, USA), in a humidified incubator (5% CO_2_, 37 °C). To induce End–MT, HUVEC were treated with 10 ng/mL TGF-β1 (Peprotech, Rocky Hill, NJ, USA), TGF-β3 (Peprotech, Rocky Hill, NJ, USA) and TNF-α (Sigma-Aldrich, St Louis, MO, USA) separately, in DMEM without serum, for 24 h and 6 days.

### 2.2. Cell Viability Assay

The effect of miRNA transfection on cell viability was determined by 3-(4,5-dimethylthiazol-2-yl)-2,5-diphenyltetrazolium bromide (MTT) assay (Vybrant MTT Cell Proliferation Assay Kit, Thermo Fisher Scientific, Waltham, MA, USA) following the manufacturer’s instructions. To this aim, cells were seeded in 96-well plates at 10^4^ cells/well in 100 μL growth medium. The MTT assay was performed on HUVECs after 24 h and 6 days exposure to TGF-β1, TGF-β3 and TNF-α, and after 24 and 48 h from miRNA over-expression. Briefly, cell medium was replaced with fresh growth medium after treatment, and 10 μL of 12 mM MTT component A (3-(4,5-dimethylthiazol-2-yl)- 2,5-diphenyltetrazolium bromide) were added to each well and left in an incubator for 4 h. Then, 100 μL of MTT component B sodium dodecyl sulfate (SDS)-hydrochloride acid (HCl) were added for a further 18 h at 37 °C. Absorbance was read at optical density (O.D.) 570 nm by a micro- plate reader.

### 2.3. Flow Cytometry

Flow cytometry was performed on HUVECs to investigate the expression of endothelial (CD31, CD146) and mesenchymal (CD44, CD105) surface markers after End-MT for 6 days. Briefly, cells were fixed by using a Fixation Kit (Beckman-Coulter, Brea, CA, USA) and stored at 4 °C. Fixed cells were incubated for 20 min using the following conjugated panel of antibodies: anti-CD31-phycoerythrin (PE) (Clone L 133.1, Beckton Dikinson BD, Franklin Lakes, NJ, USA), anti-CD146-PE (Clone s-Endo1, Biocytex), anti-CD105-phycoerythrin (PE) (Clone TEA3/17.1.1, Beckman-Coulter, Brea, CA, USA) and anti-CD44-fluorescein isothiocyanate (FITC) (Clone J.173, Beckman-Coulter, Brea, CA, USA). Negative controls were performed using appropriate conjugated irrelevant antibodies (IgG1 (Mouse)-FITC; IgG1 (Mouse)-PE, Beckman-Coulter, Brea, CA, USA). Samples were analyzed using a Navios flow cytometer equipped with 3 lasers (10-Color) for data acquisition.

### 2.4. Cell Transfection

HUVECs were seeded at a density of 1.5 × 10^5^ and 1 × 10^5^ in 12- and 24-well plates respectively, and left to adhere overnight. Transfection was performed with mirVana mimics (Thermo Fisher Scientific, Waltham, MA, USA) and Lipofectamine RNAiMAX (Thermo Fisher Scientific, Waltham, MA, USA) (1:1 ratio), according to the manufacturer’s instructions. Mimics used were: miR-30a-5p-mimic (hsa-miR-30a-5p, assay ID: MC11062, mature miRNA sequence: UGUAAACAUCCUCGACUGGAAG), miR-30d-mimic (hsa-miR-30d, assay ID: MC10756, mature miRNA sequence: UGUAAACAUCCCCGACUGGAAG) and mimic negative control (miR-NC) (10 pmol/μL). After transfection, HUVECs were processed for functional assays and RNA/protein extraction.

### 2.5. In Vitro Migration Assay

In order to evaluate the migration property of HUVECs following End–MT induction and miRNA transfection, we performed a scratch assay. Briefly, 1 × 10^5^ cells were seeded in a 24-well plate. For the End–MT study, the cell monolayer was wounded with a sterile p200 pipette tip, washed with PBS, treated with TGF-β1, TGF-β3 and TNF-α (10 ng/mL) in serum-free DMEM and incubated for additional 24–48–72 h. Cells were fixed with formalin at room temperature (rt), washed with Phosphate Buffer Saline (PBS), stained with 0.1% Crystal Violet in 25% methanol for 25 min, and air-dried. The migration property was analyzed under the phase-contrast light microscope and images were taken with a digital camera (Nikon, Tokyo, Japan). The quantification of the wounded area was performed on three fields of each treatment group and experimental time point. The wounded area was measured using the image analysis software ImageJ [20], and expressed as percentage relative to the control group at time 0.

### 2.6. Osteogenic Differentiation Assay

In order to test the effects of miRNA overexpression on the osteogenic differentiation potential, HUVECs were seeded at a density of 4 × 10^4^ cells/well on 24-well plates and left to adhere. After miRNA transfection, Optimem medium was changed with the Stem Pro Osteogenic Differentiation Kit (Thermo Fisher Scientific, Waltham, MA, USA). The miRNA transfection was repeated every three days to keep miRNA levels in cell culture. HUVECs cultured in DMEM with 10% FBS were used as controls. After 14 days, HUVECs were processed for Alizarin Red staining to detect the mineralization process, immunofluorescence, RNA and protein extraction to investigate osteogenic marker expression. For mineralization activity, cells were fixed with formalin, washed twice with PBS and stained with Alizarin Red for 30 min at rt. Images were taken under a phase-contrast light microscope and pictures were taken with a digital camera (Nikon, Tokyo, Japan). Then, Alizarin Red was dissolved by adding 10% cetylpiridinium chloride (in Na_2_HPO_4_ 10 mM, pH 7) to wells for dissolving Alizarin Red, for 15 min in the dark at rt. Calcium-bound Alizarin Red was measured by reading absorbance O.D. 570 nm by spectrophotometer.

### 2.7. RNA Extraction and Quantitative Real-Time Analysis

Total RNA was extracted from cell cultures through TRIreagent (Thermo Fisher Scientific, Waltham, MA, USA), according to the manufacturer’s instructions. Reverse Transcription was performed from one µg of total RNA in 20 µL reaction volume using the High-Capacity Reverse Transcription Kit (Thermo Fisher Scientific, Waltham, MA, USA). Real-Time PCR was carried out in a CFX Connect Real-Time PCR Detection System (BioRad Laboratories, Hercules, CA, USA) using the SYBR green mix (BioRad Laboratories) and specific couples of primers were designed using the Basic Local Alignment Tool (BLAST) from National Center for Biotechnology Information (NCBI) (purchased from Sigma-Aldrich, St Louis, MO, USA; Table 1). Each assay was performed in triplicate and target gene expression was normalized to glyceraldehyde 3-phospate dehydrogenase (GAPDH). Final results were determined by the comparative 2^^−ΔΔCt^ method and expressed as fold changes relative to untreated controls, and to control without lipofectamine in transfection experiments.

### 2.8. MicroRNA Expression Analysis

For the analysis of miRNA expression levels during End-MT, osteogenic differentiation and for the validation of miR over-expression, cDNA reverse-transcription was performed with miRNA-specific primers in the same reaction using TaqMan microRNA Assays and TaqMan microRNA Reverse Transcription Kit (Thermo Fisher Scientific, Waltham, MA, USA), following the manufacturer’s instructions. miRNA expression was performed through Real Time PCR, using TaqMan MicroRNA Assay-specific probes for each target and TaqMan Universal Master Mix II No AmpErase UNG. The reaction was carried out in CFX Connect Real-Time PCR (BioRad Laboratories, CA, USA). Each assay was executed in triplicate and target miRNA expression was normalized to non-coding small nuclear (snRNA) U6 gene. Final results were determined by the comparative 2^^−ΔΔCt^ method and expressed as fold changes relative to controls.

### 2.9. Luciferase Activity Assay

The human 3′untranslated (UTR) portion of the SLUG gene was amplified in pGEM-T Easy Vector (Promega Corporation, Madison, WI, USA), with the following primer sequences: 3′UTR FWD: GAATCTCGAGGCTGTGTAGC; 3′UTR REV: ATCTAGATGGTCAGCACAGGAG.

For the luciferase assay, HUVECs were plated at a density of 1 × 10^5^ in 96-well plates and, after 24 h, co-transfected with pmiR-GLO SLUG 3′-UTR and 10 pmol of miR-30a-5p mimics, or miR-30d mimic, or miR-negative control. Transfection was assessed with Lipofectamine RNAiMAX (Thermo Fisher Scientific, Waltham, MA, USA). After 24 h, the Firefly and Renilla luciferase activity were measured though the Dual-Glo Luciferase Assay System (Promega Corporation, Madison, WI, USA), according to the manufacturer’s instructions.

### 2.10. Immunofluorescence

For immunofluorescence, cells were fixed with cold absolute methanol for 10 min at rt, or 2% paraformaldehyde for 4 min at rt for the analysis of filamentous (F)-actin. Then, cells were permeabilized with Triton X-100 1% in PBS for an additional 10 min at rt. The blocking of non-specific binding sites was performed with bovine serum albumin (BSA) 1% in PBS for 30 min at rt. After blocking, cells were incubated with primary antibodies. For staining of F-actin, Alexa fluor 488 Phalloidin was added (1:500, Thermo Fisher Scientific, Waltham, MA, USA) for 20 min at rt, washed with PBS and counterstained with Pro Long anti-fade reagent with 4′-6-diamidino-2-phenylindole (DAPI) (Thermo Fisher Scientific, Waltham, MA, USA). For VIMENTIN (1:500; Cell Signaling), SLUG (1:100, A7, Santa Cruz Biotechnology, Dallas, TX, USA), RUNX-2 (1:100; NBP1-77461SS, Novus Biologicals, Littleton, CO, USA), SOX-9 (1:500, Abcam, Cambridge, UK) and α-SMA (1:100, Sigma-Aldrich, St Louis, MO, USA) detection, the primary antibody was incubated for 1 h at 37 °C. Samples were then washed with PBS and incubated with anti-mouse Alexa Fluor 488 and anti-rabbit Alexa Fluor 546 (Thermo Fisher Scientific, Waltham, MA, USA) secondary antibodies in 1% BSA/PBS for 1 h at 37 °C in the dark. After washing with PBS, nuclei were counterstained with DAPI. Images were acquired by a Leica DMI4000 B inverted fluorescence microscope (Leica Microsystems, Wetzlar, Germany). Quantification of SLUG, RUNX-2 and SOX-9 positive cells was performed on digitalized images randomly acquired at 40× magnification, and a minimum of 5 fields were examined for each sample. Results were expressed as percentage of nuclei positive to the target protein and expressed as percentage of positive cells/total cells.

### 2.11. Western Blot

Total cellular proteins were extracted by HUVEC using lysis buffer (0.1 M KH2PO4, pH 7.5, 1% NP-40, 0.1 mM β-glycerolphosphate, supplemented with protease inhibitor cocktail; Sigma-Aldrich, St Louis, MO, USA) and quantified spectrophometrically by the Bio-Rad Protein Assay (BioRad Laboratories, CA, USA). Thirty micrograms of proteins were separated on 12% TGX FastCast Acrylamide Solutions (BioRad Laboratories, CA, USA). Gels were imaged and the ratio of SLUG and VIMENTIN to the total protein concentration in miRNA transfection assays was measured by using Image Lab Software (BioRad Laboratories, CA, USA). Then, proteins were transferred to nitrocellulose membrane (GE Healthcare Life Sciences, Chicago, IL, USA), blocked with 5% non-fat dry milk in TBS-Tween for 1 h at rt and incubated with the following primary antibodies: VIMENTIN (1:1000; D21H3, Cell Signaling Technology, Danvers, MA, USA), SLUG (1:1000; A7, Santa Cruz Biotechnology, Dallas, TX, USA) and β-actin (1:4000; AC-74, Sigma-Aldrich, St. Louis, MO, USA) at 4 °C over night (o/n). Incubation with secondary antibody human anti-rabbit/mouse horseradish peroxidase-conjugated (GE Healthcare, Chicago, IL, USA) was performed for 1 h at rt. The protein signal was detected using Westar ηC chemiluminescent substrate (Cyanagen, Bologna, Italy).

### 2.12. Immunohistochemistry

SLUG detection by immunohistochemistry was performed on pre-existing tissue blocks obtained from archival tissues of healthy multiorgan donors and patients affected by atherosclerosis aneurysm (APP-13-01, Di.Ce. 3868-2015; St. Orsola-Malpighi Ethic Committee). All samples were anonymous and treated according to the ethical guidelines of the 1975 Declaration of Helsinki and following revisions. Immunohistochemistry was assessed using a non-biotin-amplified method (Novolink, Leica Biosystems, Wetzlar, Germany). Briefly, sections (4 μm thick) of formalin-fixed and paraffin-embedded tissues were deparaffinized and rehydrated through a series of graded ethanol and rinsed in distilled water. Endogenous peroxidase activity was blocked in 3% H_2_O_2_ in absolute methanol for 10 min at rt, antigen retrieval was performed using citrate buffer (pH 6) in autoclave (120 °C) for 20 min and, after cooling, slides were washed with Tris Buffered Saline (TBS). Sections were subsequently incubated with SLUG primary antibody (1:500; Santa Cruz Biotechnology, Dallas, TX, USA) in a moist chamber at 4 °C o/n, incubated with NovoLink Polymer for 30 min at rt and then exposed to the substrate/chromogen 3,3′-diaminobenzidine (DAB) prepared from Novocastra DAB Chromogen and NovoLink DAB buffer. Nuclei were counterstained with Mayer’s hematoxylin. Samples were dehydrated, cover slipped and observed under a light microscope using the Image Pro Plus program. Quantification of SLUG-positive cells was performed on digitalized images randomly acquired at 25× magnification, and a minimum of 5 histological sections was examined for each sample, by using the Image Pro Plus measurement tool. Results were expressed as mean of SLUG-positive areas.

### 2.13. Statistical Analysis

Each experiment was executed at least in triplicate, and all data were expressed as mean ± standard deviation (SD). The graphs and the statistical analyses were performed by GraphPad Prism 6 statistical software. The significance of differences between two experimental conditions was evaluated using the unpaired Student’s *t* test, whereas ordinary one-way analysis of variance (ANOVAs) followed by Bonferroni and Dunnett’s test was applied for multiple comparisons. Results were considered statistically significant at the 95% confidence level (*p* < 0.05).

## 3. Results

### 3.1. TGF-β1, TGF-β3 and TNF-α Induce End–MT in HUVECs

**Figure 1:**
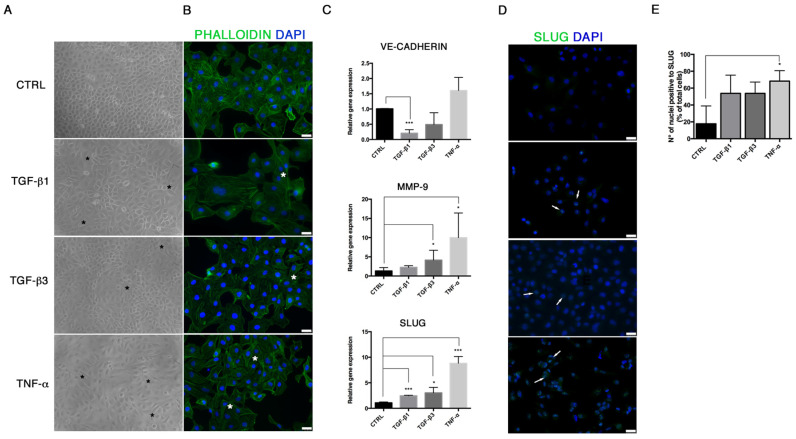
TGF-β1, TGF-β3 and TNF-α stimulate the expression of End–MT markers. (**A**) Morphological variations occurring in HUVECs after 24 h exposure to TGF-β1, TGF-β3 and TNF-α, observed under the light microscope at 20× magnification. Black stars indicate the cells that acquired a mesenchymal shape. (**B**) Cytoskeletal changes seen with F-actin immunofluorescence (20× magnification). White stars indicate cells with elongated shape. (**C**) Gene expression analysis of VE-cadherin, MMP-9 and SLUG, performed by Real-Time PCR in HUVECs after 24 h exposure to TGF-β1, TGF-β3 and TNF-α. Results are reported as fold changes relative to untreated controls. (**D**) Immunofluorescence analysis of SLUG (green spots inside nuclei, indicated by white arrows) (20× magnification). (**E**) Quantification of SLUG-positive cells expressed as percentage of cells with nuclear positivity/total cells. Scale bars = 25 μm. All data are expressed as mean ± SD of at least 3 independent experiments, and statistical analysis was performed by unpaired Student’s *t*-test; *, *p* < 0.05; ***, *p* < 0.001. MMP-9, matrix metalloproteinase 9; SLUG (SNAI2), snail family transcriptional repressor 2; TGF, transforming growth factor; TNF, tumor necrosis factor; VE-CADHERIN, vascular endothelial cadherin.

**Figure 2:**
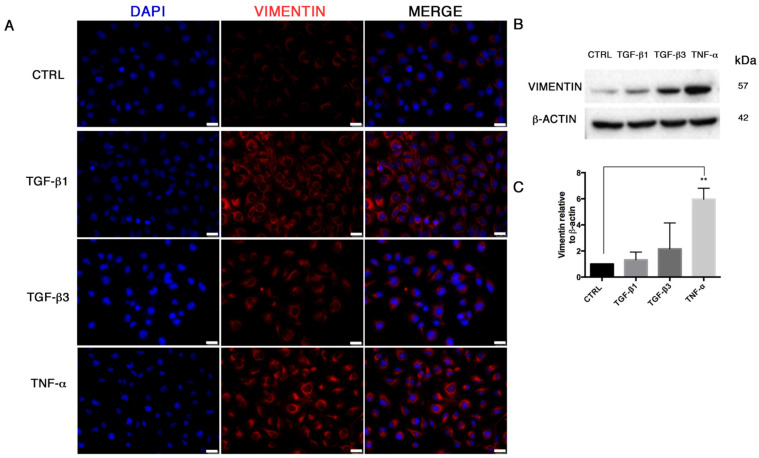
VIMENTIN expression in HUVECs during End–MT. (**A**) Immunofluorescence of VIMENTIN (red cytoplasmic extension, 40× magnification) in HUVECs after 24 h exposure to TGF-β1, TGF-β3 and TNF-α. Scale bars = 25 μm. (**B**) VIMENTIN detection in whole cell lysates assessed by Western Blot and (**C**) relative quantification to β-actin, through densitometric analysis of band intensities performed by Image J software. Results are normalized to the untreated controls. All data are expressed as mean ± SD of 3 independent experiments, and statistical analysis was performed by unpaired Student’s *t*-test, ** *p* < 0.005.

In order to study the End–MT occurring under pathological conditions in endothelial cells, we explored the effects of different soluble factors reproducing the atherosclerotic milieu in HUVECs for 24 h and 6 days. In addition to TGF-β1, the canonical inducer of End-MT, we tested TGF-β3 that is associated with the mesenchymal osteo-chondrogenic commitment, and the inflammatory cytokine TNF-α. The analysis of cell viability after these experimental conditions was measured through MTT and did not reveal cytotoxic effects both at 24 h and 6 days (Supplementary Figure S1A). After 24 h, HUVECs started an early morphological transition process from the typical cobblestone shape to a fibroblast-like morphology, mostly detectable in the TNF-α group (Figure 1A). The morphological change was reflected on the cytoskeletal rearrangements, as shown by phalloidin staining in Figure 1B. Here, the intensity stain of F-actin was broadly conserved among the treatment conditions, however the acquisition of cytoplasmic extension typical of mesenchymal shape with cytoskeletal rearrangements was highlighted, especially with TGF-β1 and TNF-α (Figure 1B; Supplementary Figure S1B). In parallel, we analyzed the expression of the endothelial gene VE-cadherin that was reduced only in cells treated with TGF-β1 (0.28 ± 0.07, *p* = 0.0003) and TGF-β3 (0.63 ± 0.3) (Figure 1C). A marked upregulation of MMP-9 mRNA (Figure 1C), associated with cell migration and matrix remodeling, and snail family transcriptional repressor 2 (SLUG, or SNAI2) mRNA (Figure 1B), was detected in HUVECs exposed to TGF-β1 (MMP-9 2.25 ± 0.21; SLUG 2.46 ± 0.07, *p* = 0.0005), TGF-β3 (MMP-9 4.14 ± 1.15, *p* = 0.048; SLUG 3.03 ± 0.6, *p* = 0.0137) and, particularly, TNF-α (MMP-9 9.98 ± 3.7, with *p* = 0.019; SLUG 8.8 ± 0.94, *p* = 0.0002). SLUG increase was further demonstrated by immunofluorescence, which revealed higher nuclear staining in all experimental conditions, especially in the presence of TNF-α (Figure 1D,E). SLUG, is a well-known marker of End–MT, broadly expressed in mesenchymal stromal cells, like VIMENTIN, whose upregulation was detected by immunofluorescence (Figure 2A) and Western blot (Figure 2B,C).

### 3.2. Persistent Inflammatory Stimulation Induces Mesenchymal Features in HUVECs

**Figure 3:**
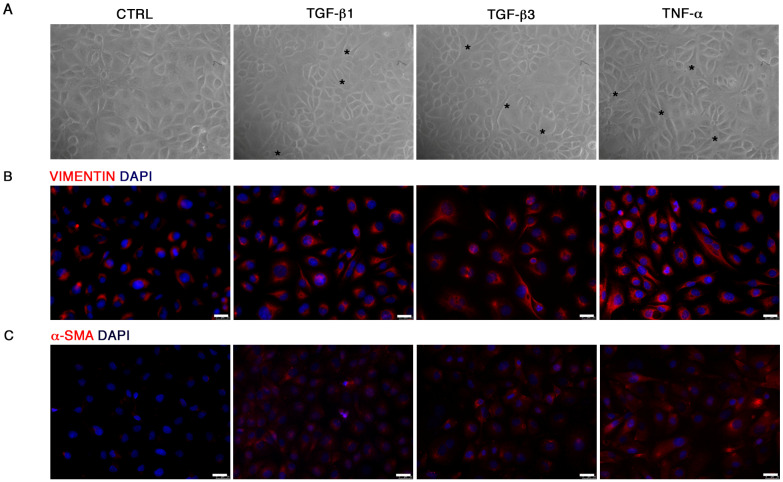
Progression of the End–MT at 6 days. (**A**) Morphological variations occurring in HUVECs after 6 days exposure to TGF-β1, TGF-β3 and TNF-α, observed under the light microscope (20× magnification). Analysis of (**B**) VIMENTIN and (**C**) α-SMA protein expression by immunofluorescence in HUVECs highlighted the gain of mesenchymal phenotype and the loss of the typical endothelial cobblestone morphology (Scale bars = 25 μm). All figures are representative of 3 independent experiments. α-SMA, smooth muscle actin.

The End–MT process was mature at longer exposure, as shown by HUVEC morphology (Figure 3A) and by immunofluorescence of VIMENTIN and α-SMA protein, emphasizing the cytoplasmic elongations typical of mesenchymal cells, especially in HUVECs exposed to TNF-α (Figure 3B,C).

**Figure 4:**
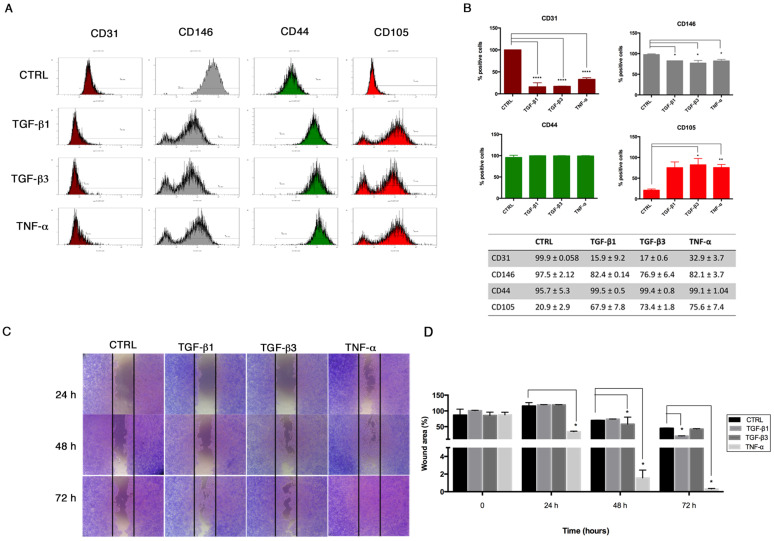
Gain of mesenchymal stem cell markers and cell migration after 6 days. (**A**) Representative flow cytometry (n = 3) of endothelial (CD31, CD146) and mesenchymal (CD44, CD105) markers in HUVECs after 6 days of exposure to TGF-β1, TGF-β3 and TNF-α. (**B**) Flow cytometry data, reported as percentage of cells positive to each marker. (**C**) Scratch assay was performed to test the migratory potential of HUVECs after treatment with TGF-β1, TGF-β3 and TNF-α. Cells were fixed and stained with Crystal after 24–48–72 h. Images are representative of three independent experiments (4× magnification). (**D**) Quantification of the wounded area, measured using ImageJ software, and expressed as percentage relative to control group at time 0. All data are expressed as mean ± SD of at least 3 independent experiments and statistical analysis was performed by unpaired Student’s *t*-test. * *p* < 0.005; ** *p* < 0.001; **** *p*< 0.0001. CD, cluster of differentiation.

Accordingly, we analyzed the immunophenotype of HUVECs after 6 days, confirming the significant decrease of the endothelial markers CD31 and CD146 expression under End–MT stimulation (Figure 4A). Here, we also observed a significant increase of the mesenchymal stem cell (MSC) marker CD105, whereas CD44 was comparable among the different conditions (Figure 4A). Results are summarized in the graphs and table in Figure 4B. The gain of a mesenchymal-like phenotype was further supported by the migratory ability, as shown by the scratch assay (Figure 4C,D). HUVECs were scratched after being exposed to TGF-β1, TGF-β3 and TNF-α, and acquired a pronounced migration to the wounded area, notably in the TNF-α condition, where the wound was completely closed at 72 h. These data confirm the phenotype plasticity of HUVECs under specific stimulation, indicating that the inflammatory response first promotes the End–MT and, later, the acquisition of a mesenchymal phenotype.

### 3.3. End–MT Provides Osteogenic Progenitors of Endothelial Origin

**Figure 5:**
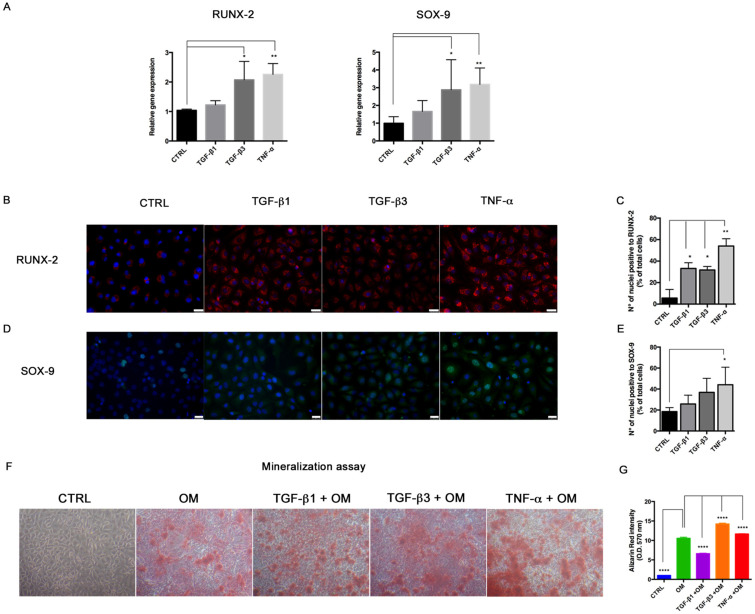
End–MT stimulates the origin of osteogenic progenitors. (**A**) Transcriptional levels of RUNX-2 and SOX-9, analyzed by Real-Time PCR in HUVECs exposed for 6 days to TGF-β1, TGF-β3 and TNF-α. Results are reported as fold changes relative to the untreated controls. Data are expressed as mean ± SD of at least 3 independent experiments, and statistical analysis was performed by unpaired Student’s *t* test. * *p* < 0.005; ** *p* < 0.001; **** *p* < 0.0001. Immunofluorescence and relative quantification of (**B**,**C**) RUNX-2 (red spots within nuclei) and (**D**,**E**) SOX-9 (green nuclei) in HUVECs exposed to TGF-β1, TGF-β3 and TNF-α for 6 days. Scale bars = 25 μm. (**F**) Alizarin Red stain performed in HUVECs exposed to TGF-β1, TGF-β3 and TNF-α for 14 days in presence of osteogenic medium (OM). (**G**) Quantification of calcium-bound Alizarin Red by absorbance measured at O.D. 570 nm by spectrophotometer. Data are expressed as mean ± SD of at least 3 independent experiments, and statistical analysis was performed with respect to HUVECs induced with osteogenic medium (OM) by ordinary one-way ANOVA followed by Dunnett’s multiple comparison test; * *p* < 0.005; ** *p* < 0.001; **** *p* < 0.0001. RUNX-2, runt-related transcription factor -2; SOX-9, SRY box transcription factor-9.

In order to investigate whether End–MT contributes to vascular calcification, we analyzed the expression of markers associated with chondrogenic and osteogenic differentiations, considering that these processes both contribute to the ectopic bone formation during atherosclerotic plaque development. At 6 days, we found an upregulation of the early markers of the osteogenic (RUNX-2: 1.2 ± 0.08 in TGF-β1, *p* = 0.053; 2.07 ± 0.36, *p* = 0.019 in TGFβ-3; 2.26 ± 0.21, *p* = 0.001 in TNF-α) and chondrogenic (SOX-9: 1.6 ± 0.36 in TGFβ-1, *p* = 0.12; 2.8 ± 1.2, *p* = 0.041 in TGFβ-3; 3.18 ± 0.5, *p* = 0.028 in TNF-α) differentiation in our End–MT model (Figure 5A). This result was confirmed by immunofluorescence, which demonstrated the increased expression of RUNX-2 (Figure 5B,C) and SOX-9 within the nucleus (Figure 5D,E), especially under TNF-α treatment.

Based on these data, we asked whether the End–MT process could trigger the gain of mineralization features in HUVECs. Thus, we performed an osteogenic differentiation assay, by culturing HUVECs in the osteogenic induction medium in combination with End–MT mediators until 14 days. As observed by the Alizarin Red stain, and relative quantification data in Figure 5F,G, mineralization occurred when HUVECs were cultured with the osteogenic medium alone. However, the addition of TGF-β3 (1.35-fold higher compared to induced sample, *p* < 0.0001) and TNF-α (1.10-fold compared to induced sample, *p* < 0.0001) to the induction medium significantly augmented the synthesis of bone matrix (Figure 5F,G).

### 3.4. SLUG Is Expressed in Human Atheroma and During In Vitro Osteogenesis

**Figure 6:**
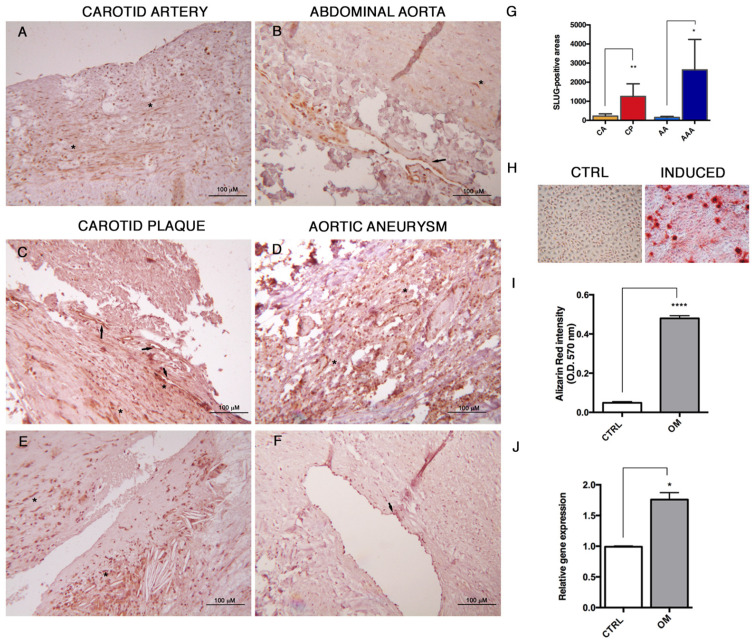
SLUG is involved in human atherosclerosis and endothelial osteogenic differentiation. (**A**) Immunohistochemistry was performed for detecting SLUG protein in human normal carotid artery, (**B**) abdominal aorta, (**C**,**E**) carotid plaque and (**D**,**F**) abdominal aortic aneurysm. Scale bars = 100 μm. (**G**) Quantification of SLUG-positive stain was performed by Image Pro Plus software as the sum of positive areas and reported as mean ± SD. Abbreviations used in the graph: CA: carotid artery; CP: carotid plaque; AA: abdominal aorta; AAA: abdominal aortic aneurysm. (**H**) Mineralization assay performed on HUVECs after 14 days of exposure to osteogenic induction medium, and stained with Alizarin Red. (**I**) Quantification of calcium-bound Alizarin Red by absorbance measured at O.D. 570 nm by spectrophotometry. Data were normalized to control in DMEM 10% FBS. (**J**) Transcriptional levels of SLUG in HUVECs differentiated toward the osteogenic lineage after induction with specific medium for 14 days. Results are reported as fold changes relative to controls in DMEM 10% FBS. All data are expressed as mean ± SD of at least 3 independent experiments, and statistical analysis was performed by unpaired Student’s *t* test. *, *p* < 0.05; **, *p* < 0.001; ****, *p* < 0.0001.

In order to detect the occurrence of End–MT in vivo, we studied the expression of SLUG in tissue specimens from human carotid plaques and abdominal aortic aneurysms, compared to normal, healthy arterial tissue sections. In Figure 6A,B, it is possible to see focal and weak positivity in medial SMCs, and moderate to strong positivity in endothelial cells lining vasa vasorum. Conversely, in carotid plaques (Figure 6C,E) and aneurysmal aortas (Figure 6D,F), the SLUG positivity was intense and diffuse, especially in cells lining the lumen of neo-vessels and among inflammatory cell infiltrates. The sum of areas of SLUG cell positivity was measured and reported as mean ± standard deviation in Figure 6G, evidencing a significant increase of SLUG protein in carotid plaques (CP) and abdominal aortic aneurysms (AAA) in comparison to the respective healthy controls. These data suggest the involvement of SLUG in both the physiological and pathological remodeling process, and its possible participation to atherosclerosis progression. In order to study the contribution of SLUG to the mesenchymal differentiation, HUVECs were exposed to osteogenic induction medium for 14 days (Figure 6H,I). After treatment, SLUG expression resulted increased in HUVECs primed to osteogenesis in comparison to controls, as shown by its increased transcript levels in Figure 6J (1.8-fold increase, *p* = 0.018). These results might support an association between End–MT and vascular calcification.

### 3.5. miR-30a-5p and miR-30d Are Downregulated during End–MT

**Figure 7:**
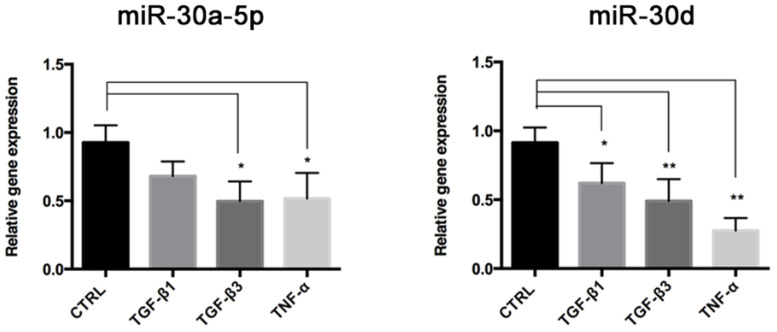
miR-30a-5p and miR-30d are downregulated during End–MT. Relative expression of miR-30a-5p and miR-30d in HUVECs after 6 days of exposure to TGF-β1, TGF-β3 and TNF-α. Results are reported as fold changes relative to the untreated group. All data are expressed as mean ± SD of at least 3 independent experiments, and statistical analysis was performed by unpaired Student’s *t* test. * *p* < 0.005; ** *p* < 0.001. miR-NC, miRNA negative control.

We investigated the involvement of miR-30a-5p and miR-30d during End–MT and osteogenic differentiation, by analyzing their expression levels in HUVECs exposed to End–MT inducers for 6 days. Expression of miR-30a-5p and miR-30d was lower in HUVECs that underwent End–MT (Figure 7). In particular, miR-30a-5p decrease was significant in cells exposed to TGF-β3 (0.49 ± 0.08, *p* = 0.018) and TNF-α (0.51 ± 0.11, *p* = 0.001), whereas miR-30d was reduced in all experimental groups (0.62 ± 0.08, *p* = 0.027 in TGF-β1; 0.49 ± 0.07, *p* = 0.004 in TGF-β3; 0.27 ± 0.06, *p* = 0.002 in TNF-α). These data suggest that these miRNAs can be involved during the End–MT process.

### 3.6. Effects of miR-30a-5p and miR-30d Over-Expression in HUVECs

**Figure 8:**
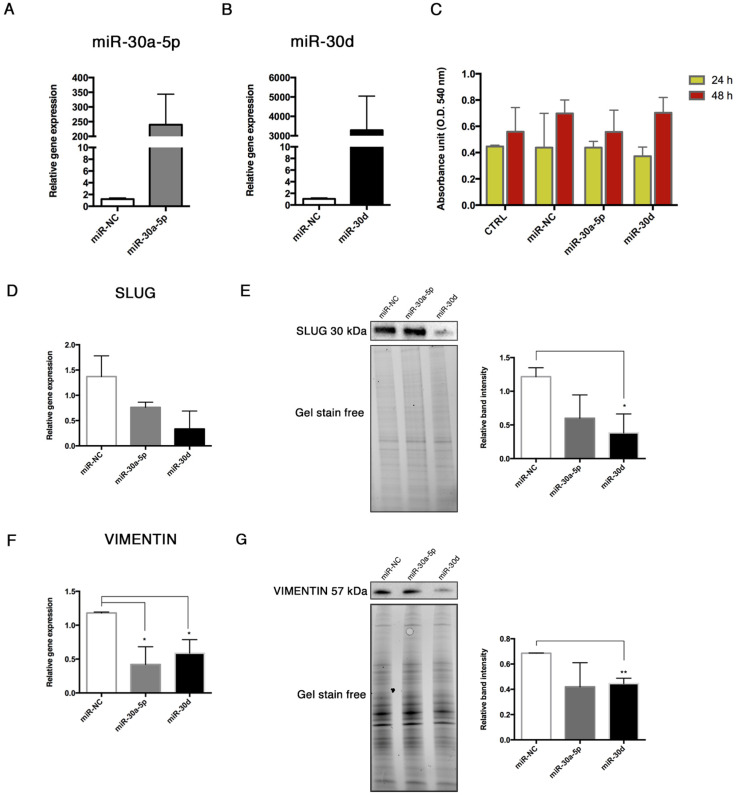
miR-30a-5p and miR-30d over-expression in HUVEC. Over-expression of (**A**) miR-30a-5p and (**B**) miR-30d in HUVECs transfected through Lipofectamine RNAiMAX. Results are reported as fold changes relative to control. (**C**) Effects of miR-30a-5p and miR-30d mimics on HUVEC viability at 24 and 48 h, evaluated through the MTT assay. Analysis of SLUG (**D**) mRNA and (**E**) protein, analysis of VIMENTIN (**F**) mRNA and (**G**) protein. Western Blot bands were normalized to total protein amount through gel stain free using the ImageLab Software. Real-Time results are reported as fold changes to control. All data are expressed as mean ± SD of 4 independent experiments, and statistical analysis was performed by unpaired *t* test. * *p* < 0.05; ** *p* < 0.01. miR-NC, miRNA negative control; MTT, 3-(4,5-dimethylthiazol-2-yl)-2,5-diphenyltetrazolium bromide.

To investigate whether miR-30a-5p and miR-30d regulate the End–MT, we overexpressed these miRNAs in HUVECs. We preliminarily verified the effects of the transfection, observing the upregulation of miR-30a-5p and miR-30d of 239 ± 103.9, and 3289 ± 1757 -folds respectively, in transfected cells compared to controls after 48 h (Figure 8A,B). Then, we explored possible effects of miRNA transfection on cell viability, which did not result affected, as shown by the MTT assay (Figure 8C). The expression levels of the main End–MT markers were analyzed after 48 h over-expression. SLUG mRNA displayed a decreasing trend (Figure 8D), whereas a significant downregulation of its protein was observed after miR-30d over-expression (0.38 ± 0.16, *p* = 0.032) (Figure 8E). VIMENTIN showed a significant downregulation of mRNA levels with both miRNAs (0.42 ± 0.15, *p* = 0.03 in miR-30a-5p over-expressing HUVECs; 0.58 ± 0.11, *p* = 0.028 in miR-30d over-expressing HUVECs) (Figure 8F), and a reduced protein expression mainly with miR-30d (Figure 8G).

**Figure 9:**
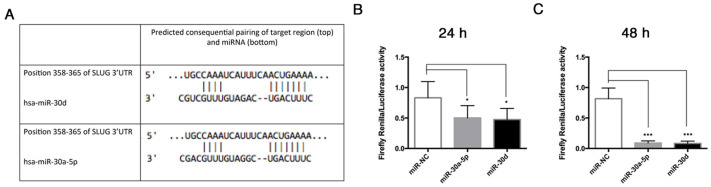
miR-30a-5p and miR-30d target SLUG gene. (**A**) Identification of the putative binding sites of miR-30a-5p and miR-30d in the 3′-UTR of SLUG, as reported by TargetScan analysis. (**B**) 24 h and (**C**) 48 h analysis of Luciferase relative activity in HUVECs co-transfected with pmiR-GLO-SLUG 3′-UTR and miR-30a-5p, miR-30d, or miR-NC. Luciferase activity was normalized to Renilla luciferase activity. The resulting data in HUVECs co-transfected with pmiR-GLO-SLUG 3′-UTR and miR-30a-5p, miR-30d, or miR-NC, were normalized to HUVECs co-transfected with empty vector and the respective oligonucleotides. Results are expressed as mean ± SD of four replicates, and statistical analysis was performed by unpaired *t* test. * *p* < 0.005, *** *p* < 0.001. UTR: untranslated region.

The bioinformatic analysis carried out by the TargetScan algorithm identified SLUG among predicted targets of miR-30a-5p and miR-30d, whose binding sequence was found in the 3′-UTR (Figure 9A). To demonstrate whether miR-30a-5p and miR-30d directly bind the 3′-UTR of SLUG, we assessed a luciferase reporter assay. To this aim, a portion of SLUG 3′UTR, containing two binding sites for miR-30a-5p and miR-30d, was cloned in the pmiR-GLO vector downstream of a firefly luciferase reporter gene. Then, HUVECs were co-transfected with this vector and miR-30a-5p, miR-30d, or miR-NC. After 24 h, we found that co-transfection of pmiR-GLO SLUG 3′UTR with miR-30a-5p in HUVECs reduced the luciferase activity by 39% (*p* = 0.014) in comparison to HUVECs co-transfected with miR-NC. Similarly, co-transfection of pmiR-GLO SLUG 3′UTR with miR-30d in HUVECs reduced the luciferase activity by 43% (*p* = 0.0073) in comparison to HUVECs co-transfected with miR-NC (Figure 9B). After 48 h, the relative luciferase activity decreased to 88% (*p* = 0.0004) in HUVECs co-transfected with pmiR-GLO SLUG 3′UTR and miR-30a-5p, and to 89% (*p* = 0.0004) in HUVECs co-transfected with pmiR-GLO SLUG 3′UTR with miR-30d, compared to HUVECs co-transfected with miR-NC (Figure 9C). These data indicate that both miR-30a-5p and miR-30d directly bind to 3′-UTR of SLUG mRNA.

### 3.7. Effects of miR-30a-5p and miR-30d Gain of Function on the Osteogenic Differentiation Process

**Figure 10:**
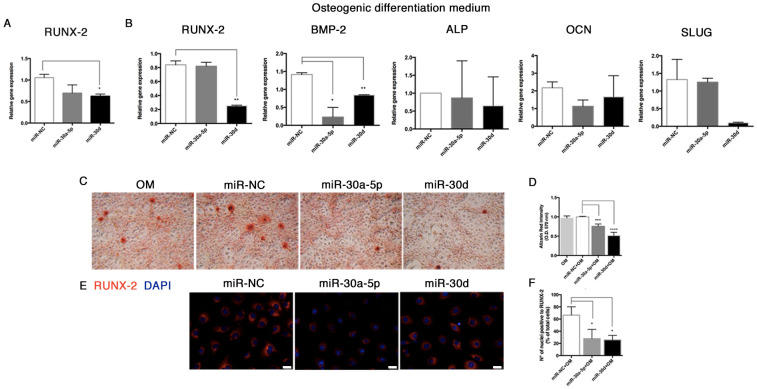
miR-30a-5p and miR-30d modulate the osteogenic differentiation potential in HUVECs. (**A**) RUNX-2 relative expression in HUVECs at 48 h post-miRNA transfections. (**B**) Gene expression analysis (RUNX-2, BMP-2, ALP, OCN, SLUG) in HUVECs after miRNA over-expression during 14-day exposure to osteogenic induction medium. (**C**) Alizarin red stain of HUVECs after miRNA over-expression during osteogenic induction for 14 days, and (**D**) relative quantification, performed by reading the absorbance at O.D. 570 nm by spectrophotometer. Images are representative of at least three independent experiments (40× magnification). Results were normalized to control cells cultured in DMEM. (**E**) Immunofluorescence of RUNX-2 in HUVECs after miRNA over-expression during osteogenic induction for 14 days, and (**F**) relative quantification expressed as percentage of positive nuclei on total cell number. Scale bars = 25 μm. OM: osteogenic medium. All data are expressed as mean ± SD of 4 independent experiments, and statistical analysis was performed between miRNA-mimic transfected cells and miR-NC group, by unpaired Student’s *t*-test. *, *p*< 0.05; **, *p* < 0.01; ***, *p* < 0.001; ****, *p* < 0.0001. BMP-2, bone morphogenetic protein-2; ALP, alkaline phosphatase; OCN, osteocalcin.

In order to explore the effect of miR-30a-5p and miR-30d on the calcification process in vitro, we preliminarily analyzed the expression of RUNX-2 in HUVECs transfected with miR-30a-5p-mimic and miR-30d-mimic. Here, we observed a significant downregulation of RUNX-2 at 48 h post-transfection (0.69 ± 0.10 with miR-30a-5p-mimic, and 0.62 ± 0.03 with miR-30d-mimic, *p* = 0.002) (Figure 10A).

miRNA over-expression for 48 h was performed in HUVECs exposed to osteogenic medium for 14 days. After treatment, we analyzed the mRNA expression of the key osteogenic markers, observing a downregulation trend for RUNX-2 (0.25 ± 0.05 in miR-30d over-expressing HUVECs, *p* = 0.047), BMP-2 (0.23 ± 0.19 in miR-30a-5p over-expressing HUVECs, *p* = 0.0256; 0.84 ± 0.05 in miR-30d over-expressing HUVECs, *p* = 0.0038). Alkaline phosphatase (ALP) and osteocalcin (OCN) (Figure 10B) were slightly modulated. A decreasing trend of SLUG was also recorded in transfected cells.

Interestingly, a significant lowering of the mineralization activity was reported in HUVECs transfected with both miRNAs, as shown by Alizarin Red stain and relative quantification (0.75 ± 0.03, *p* = 0.0002 with miR-30a-5p; 0.51 ± 0.05, *p* < 0.0001 with miR-30d) (Figure 10C,D). To further confirm these findings, we investigated the protein levels of RUNX-2 by immunofluorescence (Figure 10E), where a significant reduction of nuclear staining by 25% (*p* = 0.03) was reported in HUVECs transfected with miR-30a-5p-mimic, and by 27% (*p* = 0.01) was reported in HUVECs transfected miR-30d-mimic (Figure 10F).

## 4. Discussion

In the present study, we investigated the involvement of miR-30a-5p and miR-30d during End–MT and vascular calcification. To this aim, we firstly established a cell model for studying End–MT and its potential association with the in vitro osteogenic process. We therefore explored the endothelial cell plasticity and phenotype transition in the presence of growth factors and cytokines mimicking the inflammatory microenvironment expected to be present during the development of the atheromatous plaque. During End–MT, endothelial cells lose their typical cobblestone shape and markers, switching to a MSC phenotype, including markers, cytoskeleton rearrangements and properties, i.e., migration, multilineage differentiation. End–MT occurs under vascular injury, representing an intermediate step in atherosclerosis and calcification [21]. We demonstrated that TGF-β1, TGF-β3 and TNF-α reproduce the inflammatory setting in vitro and induce End–MT in HUVECs, which acquire morphology (evidenced by F-actin staining), markers (MMP-9, SLUG, VIMENTIN, CD105) and motility, typical of MSCs. In parallel, we detected the loss of endothelial markers in all experimental conditions (CD31, CD146). VE-CADHERIN expression resulted affected only in TGF-β1 and TGF-β3, whereas it was increased in cells treated with TNF-α, indicating a possible compensatory response to keep the endothelial barrier functionality.

Interestingly, we also detected the upregulation of the transcriptional factors RUNX-2 and SOX-9, commonly associated with the osteo/chondrogenic lineage. We further demonstrated that the combination of TGF-β3 and TNF-α with osteogenic induction medium significantly increased the mineralization activity in HUVECs. These results suggest the ability of transitional endothelial cells to become a significant contributor of specialized cells able to release pro-calcific matrix. Since vascular calcification is currently considered a stem cell-driven process, endothelial cells seem to acquire a novel and dynamic role during vascular remodeling and disease progression. Thus, End–MT represents a novel and promising target for preventing the pathological bone formation. Comparing all data, we noticed that TNF-α and TGF-β3 were more effective than TGF-β1 at triggering endothelial cell plasticity versus a mesenchymal/osteogenic phenotype. These data confirm the pivotal role of inflammation to the vascular remodeling and calcification process, and also suggest the contribution of the TGF-β3 signaling to the atherosclerosis complications.

Once established this in vitro model of End–MT, we investigated the role of miR-30a-5p and miR-30d as possible modulators of endothelial cell behavior. Indeed, miRNAs recently emerged as novel and intriguing regulators of many biological processes, including End–MT, through their ability to target genes that are critical to endothelial cell trans-differentiation [22]. As a first step, we analyzed the expression levels of miR-30a-5p and miR-30d, members of the miR-30 family that controls the development and biology of bone, adipose tissue and blood vessels [23]. We found that these miRNAs were both downregulated in End–MT, especially in TGF-β3 and TNF-α conditions. This result strengthens the hypothesis on a direct link between the osteo/chondrogenic commitment, inflammation and End–MT. The analysis performed through TargetScan identified that VIMENTIN and SNAI1 are predicted targets of miR-30a-5p and miR-30d [15], thus we explored the interactions between these miRNAs and some End–MT mediators by performing gain-of-function experiments in HUVECs. The over-expression of miR-30a-5p and miR-30d in HUVECs was associated with the downregulation of SLUG, notably with miR-30d. Interestingly, we observed a significant reduction of the relative luciferase activity in HUVECs co-transfected with the pmiR-Glo 3′UTR SLUG and miR-30a-5p, and miR-30, after 24 and 48 h. These data demonstrated that a direct interaction exists between SLUG and both miRNAs, therefore SLUG is a direct target of miR-30a-5p and miR-30d. We also observed a significant decrease of VIMENTIN following miRNA over-expression, and this result was in accordance with literature studies that have already investigated that VIMENTIN is a direct target of miR-30a-5p [24,25]. These data imply that miR-30a-5p and miR-30d may target the pathological differentiation of endothelial cells. The role of the miR-30 family in End–MT has been poorly investigated. More data are available with regard to EMT. Chung et al. demonstrated the inhibitory role of miR-30a-5p on EMT by increasing the tight-junction claudin-5 in human upper tract urothelial carcinoma cells [16]. Further, miR-30a-5p targets the ROR1, the orphan like receptor tyrosine kinase (RTK), reducing EMT and metastasis formation in triple-negative breast cancer [26]. However, data on endothelial cells are few. Jiang et al. observed that miR-30a stimulates arteriolar branching [27], whereas another work on miR-30b showed its inhibitory role on capillary morphogenesis acting on TGF-β2 signaling [28]. Thus, members of the same miRNA family may exploit distinct functions.

Considering that End–MT is strongly connected with the osteogenic differentiation process and atherosclerotic calcification, we further explored this issue. The members of the miR-30 group can regulate the osteogenic process, indeed their over-expression regulated the BMP-2-induced osteogenic process by targeting RUNX-2 and Smad1 in mouse osteoblasts and bone marrow mesenchymal stem cells [29]. Moreover, it was shown that Bone Morphogenetic Protein 2 (BMP-2) was able to downregulate miR-30b and miR-30c, consequently increasing RUNX-2 expression in a model of human coronary artery SMCs [30]. A recent work also demonstrated that miR-30b is able to reduce vascular calcification in vivo [31]. Another study demonstrated that miR-30a-5p levels were decreased in serum of atherosclerotic patients, and the same research explored the role of miR-30a-5p in a monocyte–endothelial cell co-culture system, showing that miRNA overexpression in the human monocyte cell line THP-1 cells protects endothelial cells from apoptosis [32]. Our recently published study reported that miR-30a-5p and miR-30d are differentially expressed in carotid plaques exhibiting different calcification patterns. In addition, the expression levels of miR-30a-5p and miR-30d were reduced in endothelial cells after osteogenic differentiation [14]. These preliminary observations and previous studies, which elucidated the involvement of the miR-30 family in MSC osteogenic differentiation, prompted us to study miR-30a-5p and miR-30d in endothelial cells under osteogenic conditions. To this purpose, we transfected HUVECs with miR-30a-5p-mimic and miR-30d-mimic during osteogenic induction, for 14 days. At the end of treatment, we noticed a significant lowering of the mineralization ability in transfected HUVECs, in accordance with the reduced expression of RUNX-2 and BMP-2 genes.

In this study, we also highlight the relevance of SLUG to atheroma calcification, whose involvement during arterial calcification has not been elucidated yet. Sànchez-Duffhues et al. demonstrated that SLUG is involved in endothelial cell-mediated calcification [33]. Here, we report that SLUG is highly expressed in human atherosclerosis, both at the aortic and carotid level, as well as in vitro when HUVECs differentiated into osteoprogenitors. These data implicate the involvement of SLUG during the pathological remodeling occurring in atherosclerosis and calcification processes, providing novel cues for investigating the atherosclerotic calcification and the search for new therapeutic approaches.

To the best of our knowledge, this is the first study addressing the role of miR-30a-5p and miR-30d in endothelial cells with respect to the End–MT and calcification process. We showed that End–MT transformation is tightly associated with the vascular bone formation under the inflammatory milieu and represents a potential clinically relevant target. Our results, in accordance with the literature data, support a negative correlation between the investigated miRNA and the atherosclerotic calcification. miRNAs are attractive candidates for promising therapeutic approaches aimed at controlling the disease progression, by regulating the main upstream mechanisms, as well as possible stage-specific markers. The present study can therefore be preliminary to future investigations for elucidating the role of miR-30a-5p and miR-30d, novel signaling pathways connected with atherosclerosis and calcification and the possible delivery approach in the light of their utilization in the clinical practice.

## 5. Conclusions

In the present study, we demonstrated that two members of the miR-30 family, miR-30a-5p and miR-30d, are deregulated during End–MT and possess a key role during this process. We established an in vitro End–MT model in the presence of soluble factors belonging to the TGF family and to the inflammatory cytokines. We observed that TGF-β1, TGF-β3 and TNF-α stimulate the occurrence of End–MT, a phenotype switch that triggers different pathological vascular changes, and the increase of mineralization activity. Under these conditions, miR-30a-5p and miR-30d were downregulated; conversely, their over-expression was associated with a decreased expression of SLUG, VIMENTIN and with a less prominent osteogenic differentiation in HUVECs. Altogether, our data support the involvement of miR-30a-5p and miR-30d in the endothelial cell plasticity toward the osteogenic phenotype, but further investigations are needed to unveil this regulatory axis.

## Figures and Tables

**Table 1:** List of primer sequences used for Real-Time PCR.

Gene Name	Primer Sequences
ALP	FWD GGGCTCCAGAAGCTCAACAC REV GTGGAGCTGACCCTTGAGCAT
GAPDH	FWD AATGGGCAGCCGTTAGGAAA REV AGGAGAAATCGGGCCAGCTA
MMP-9	FWD GAACCAATCTCACCGACAG REV GCCACCCGAGTGTAACCAT
OCN	FWD CACCGAGACACCATGAGAGC REV CTGCTTGGACAAAGGCTGC
RUNX-2	FWD TGATGACACTGCCACCTCTGA REV GCACCTGCCTGGCTCTTCT
SLUG	FWD TTCAACGCCTCCAAAAAGCC REV GATGGGGCTGTATGCTCCTG
SOX-9	FWD AGTACCCGCACCTGCACAAC REV CGCTTCTCGCTCTCGTTCAG
VE-CADHERIN	FWD GATGCAGAGGCTCATGATGC REV CTTGCGACTCACGCTTGACT
VIMENTIN	FWD ATCGATGTGGATGTTTCCAA REV TTGTACCATTCTTCTGCCTC

ALP, alkaline phosphatase; GAPDH, glyceraldehyde 3-phospate dehydrogenase; MMP-9, matrix metalloproteinase 9; OCN, osteocalcin; RUNX-2, runt-related transcription factor 2; SLUG (SNAI2), snail transcriptional repressor 2; SOX-9, SRY box transcription factor 9; VE-CADHERIN, vascular endothelial cadherin.

## Data Availability

All relevant data are available within the manuscript and supplementary materials.

## Supplementary Materials

The following are available online at https://www.mdpi.com/2218-273X/11/2/226/s1, Figure S1: End-MT in HUVEC.
