# Supplementary material for: Involvement of miR-30a-5p and miR-30d in Endothelial to Mesenchymal Transition and Early Osteogenic Commitment under Inflammatory Stress in HUVEC
Source: Biomolecules. 2021 Feb 5;11(2):226. doi: 10.3390/biom11020226 (PMC7915105; doi:10.3390/biom11020226)
Supplement: Supplementary file 1 [file biomolecules-11-00226-s001.pdf]

Supplementary File

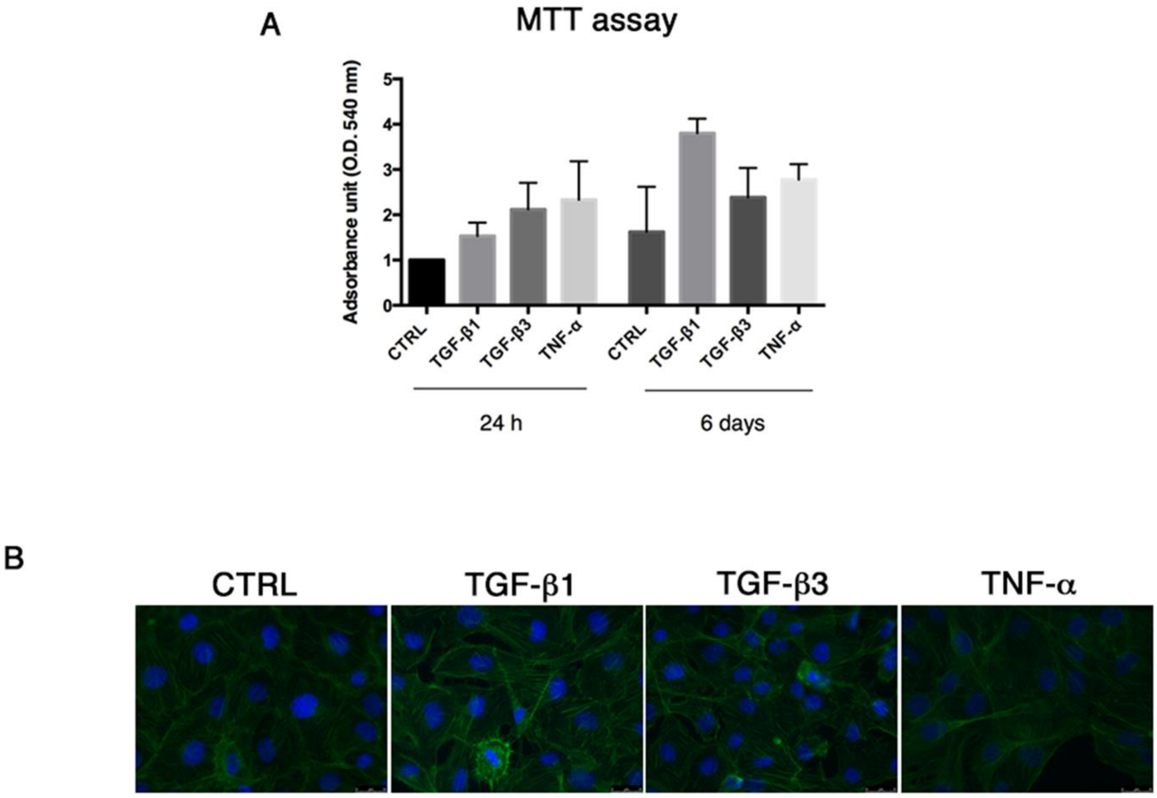

Figure S1. End-MT in HUVEC. (**A**) Analysis of HUVEC viability after 24 h and 6 days exposure to TGF-β1, TGF-β3 and TNF-α. (**B**) Cytoskeletal changes seen with F-actin immunofluorescence (40× magnification).
